# ^124^I-HuCC49deltaC_H_2 for TAG-72 antigen-directed positron emission tomography (PET) imaging of LS174T colon adenocarcinoma tumor implants in xenograft mice: preliminary results

**DOI:** 10.1186/1477-7819-8-65

**Published:** 2010-08-06

**Authors:** Peng Zou, Stephen P Povoski, Nathan C Hall, Michelle M Carlton, George H Hinkle, Ronald X Xu, Cathy M Mojzisik, Morgan A Johnson, Michael V Knopp, Edward W Martin, Duxin Sun

**Affiliations:** 1 Division of Pharmaceutics, College of Pharmacy, The Ohio State University, Columbus, Ohio, 43210, USA; 2 Current Address: Department of Pharmaceutical Sciences, College of Pharmacy, The University of Michigan, Ann Arbor, Michigan, 48109, USA; 3 Division of Surgical Oncology, Department of Surgery, Arthur G. James Cancer Hospital and Richard J. Solove Research Institute and Comprehensive Cancer Center, The Ohio State University, Columbus, Ohio, 43210, USA; 4 Department of Radiology, The Ohio State University, Columbus, Ohio, 43210, USA; 5 Division of Pharmacy Practice, College of Pharmacy, The Ohio State University, Columbus, Ohio, 43210, USA; 6 Department of Biomedical Engineering, The Ohio State University, Columbus, Ohio, 43210, USA

## Abstract

**Background:**

^18^F-fluorodeoxyglucose positron emission tomography (^18^F-FDG-PET) is widely used in diagnostic cancer imaging. However, the use of ^18^F-FDG in PET-based imaging is limited by its specificity and sensitivity. In contrast, anti-TAG (tumor associated glycoprotein)-72 monoclonal antibodies are highly specific for binding to a variety of adenocarcinomas, including colorectal cancer. The aim of this preliminary study was to evaluate a complimentary determining region (CDR)-grafted humanized C_H_2-domain-deleted anti-TAG-72 monoclonal antibody (HuCC49deltaC_H_2), radiolabeled with iodine-124 (^124^I), as an antigen-directed and cancer-specific targeting agent for PET-based imaging.

**Methods:**

HuCC49deltaC_H_2 was radiolabeled with ^124^I. Subcutaneous tumor implants of LS174T colon adenocarcinoma cells, which express TAG-72 antigen, were grown on athymic Nu/Nu nude mice as the xenograft model. Intravascular (i.v.) and intraperitoneal (i.p.) administration of ^124^I-HuCC49deltaC_H_2 was then evaluated in this xenograft mouse model at various time points from approximately 1 hour to 24 hours after injection using microPET imaging. This was compared to i.v. injection of ^18^F-FDG in the same xenograft mouse model using microPET imaging at 50 minutes after injection.

**Results:**

At approximately 1 hour after i.v. injection, ^124^I-HuCC49deltaC_H_2 was distributed within the systemic circulation, while at approximately 1 hour after i.p. injection, ^124^I-HuCC49deltaC_H_2 was distributed within the peritoneal cavity. At time points from 18 hours to 24 hours after i.v. and i.p. injection, ^124^I-HuCC49deltaC_H_2 demonstrated a significantly increased level of specific localization to LS174T tumor implants (p = 0.001) when compared to the 1 hour images. In contrast, approximately 50 minutes after i.v. injection, ^18^F-FDG failed to demonstrate any increased level of specific localization to a LS174T tumor implant, but showed the propensity toward more nonspecific uptake within the heart, Harderian glands of the bony orbits of the eyes, brown fat of the posterior neck, kidneys, and bladder.

**Conclusions:**

On microPET imaging, ^124^I-HuCC49deltaC_H_2 demonstrates an increased level of specific localization to tumor implants of LS174T colon adenocarcinoma cells in the xenograft mouse model on delayed imaging, while ^18^F-FDG failed to demonstrate this. The antigen-directed and cancer-specific ^124^I-radiolabled anti-TAG-72 monoclonal antibody conjugate, ^124^I-HuCC49deltaC_H_2, holds future potential for use in human clinical trials for preoperative, intraoperative, and postoperative PET-based imaging strategies, including fused-modality PET-based imaging platforms.

## Background

The origin of positron imaging dates back to the early 1950's [1], culminating in the development of positron emission tomography (PET) and its subsequent evolution over the last 40 years [1-4]. The clinical application of PET-based imaging strategies to the field of oncology has had a significant impact upon the care of cancer patients [5-11]. Therefore, the development and selection of the most appropriate and specific radiotracer for PET-based imaging is critical to its success in oncology [12-15].

^18^F-fluorodeoxyglucose (^18^F-FDG) is currently the most widely used radiotracer for PET-based imaging strategies [16]. In this regard, ^18^F-FDG-PET-based imaging is considered state-of-the-art for the diagnostic imaging, staging, and follow-up of a wide variety of malignancies, including colorectal cancer [10,11]. However, there are several intrinsic limitations related to the use of ^18^F-FDG-PET imaging that remain a challenge and a concern to those involved in the care of cancer patients [6-9,16-24]. First, false positive results can occur with ^18^F-FDG-PET imaging in the presence of any pathologic conditions in which there is a high rate of glucose metabolism, such as inflammatory or infectious processes. Second, false negative results can occur with ^18^F-FDG-PET imaging secondary to poor avidity of ^18^F-FDG to certain tumor types and secondary to impaired uptake of ^18^F-FDG in patients with elevated blood glucose levels. Third, due to system resolution limitations, ^18^F-FDG-PET imaging is generally limited in its ability to detect small-volume, early-stage primary disease or to detect microscopic disease within the lymph nodes. Fourth, ^18^F-FDG-PET imaging can produce either false positive or false negative results secondary to the normal physiologic accumulation of ^18^F-FDG within certain tissues with an elevated level of glucose metabolism (most striking in the brain and heart, and to a lesser degree in the mucosa and smooth muscle of the stomach, small intestine and colon, as well as in liver, spleen, skeletal muscle, thyroid, and brown fat) and secondary to the excretion and accumulation of ^18^F-FDG within the urinary tract (kidneys, ureters, and bladder). Overall, these factors have a negative impact on optimizing the specificity and sensitivity of ^18^F-FDG-PET for accurate diagnostic cancer imaging [6-9,16-24].

A PET-based imaging approach that specifically targets the cancer cell environment would clearly have a significant potential advantage for improving the accuracy of diagnostic cancer imaging over that of the more nonspecific nature of ^18^F-FDG. In that regard, tumor-associated glycoprotein-72 (TAG-72) is a mucin-like glycoprotein complex that is overexpressed by many adenocarcinomas, including colorectal, pancreatic, gastric, esophageal, ovarian, endometrial, breast, prostate, and lung [22,24-27]. Such overexpression of TAG-72 is noted in up to approximately 90% of these various adenocarcinomas [24]. In xenograft mice bearing subcutaneous tumor implants of the TAG-72-expressing human colon adenocarcinoma cell line, LS174T [27-29], anti-TAG-72 monoclonal antibodies have been shown to accumulate up to 18-fold higher in LS174T tumor implants than in normal tissues [25,30,31]. Over the last 25 years, our group at The Ohio State University, as well as others, have evaluated a variety of radioiodine labeled anti-TAG-72 monoclonal antibodies for tumor-specific antigen targeting at the time of surgery for known primary, recurrent, and metastatic disease, as well as for targeting occult disease and affected lymph nodes in colorectal cancer patients [22,24,32-59]. Most recently, we have evaluated the complimentary determining region (CDR)-grafted humanized C_H_2-domain-deleted anti-TAG-72 monoclonal antibody, HuCC49deltaC_H_2 [60-63], radiolabeled with iodine-125 (^125^I), for intraoperative tumor detection of colorectal cancer in both a preclinical xenograft mouse model and in a human clinical trial [22,24,57-59]. Collectively, our experience with radiolabeled anti-TAG-72 monoclonal antibodies in combination with a handheld gamma detection probe has clearly shown that this technology provides the surgeon with real-time intraoperative information for more precise tumor localization and resection and has demonstrated improved long-term patient survival after surgery [22,24].

Because of the drawbacks of using ^125^I as the radioiodine label for anti-TAG-72 monoclonal antibodies, including the extremely long physical half-life of ^125^I of approximately 60 days (which generates handling, storage, and disposal issues within the operating room environment and in the surgical pathology department) and the inability of ^125^I to allow for diagnostic imaging capabilities, other radionuclides have been sought for use with anti-TAG-72 monoclonal antibodies. One such alternative is iodine-124 (^124^I) [64]. In this regard, ^124^I is a positron emitting radionuclide that has a physical half-life of approximately 4.2 days, for which its positron emitting properties makes it well-suited for PET-based imaging and for which its shorter physical half-life simplifies the handling, storage, and disposal issues. Therefore, the aim of this preliminary study was to evaluate ^124^I-HuCC49deltaC_H_2 as an antigen-directed and cancer-specific targeting agent for PET-based imaging.

## Methods

### Tissue culture and reagents

Cell culture medium (DMEM), fetal bovine serum (FBS), trypsin, and other tissue culture materials were purchased from Invitrogen (Carlsbad, California).

The human colon adenocarcinoma cells (LS174T) [27-29] were purchased from American Type Culture Collection (ATCC) (Manassas, VA). LS174T cells were cultured in DMEM (10% FBS, 1% penicillin/streptomycin) at 37°C in a humidified atmosphere with 5% CO_2 _and with the medium changed daily. LS174T cells were divided weekly. LS174T cells were trypsinized, collected, and washed with PBS, and resuspended in DMEM (10% FBS) for the subculture process. LS174T cells were stored in DMEM (20% FBS, 10% DMSO) in liquid N_2_.

DOTA chelated HuCC49deltaC_H_2 antibody was supplied by Dr. Jeffrey Schlom (Laboratory of Tumor Immunology and Biology, National Cancer Institute, National Institutes of Health, Bethesda, MD).

Phosphate buffered ^18^F-FDG (200 MBq/ml) was supplied by IBA Molecular (Dulles, VA).

### Iodination (^124^I) of HuCC49deltaC_H_2

#### Iodogen-coated Vials

Iodogen (1,3,4,6-tetrachloro-3α-6α-diphenylglycouril) (Pierce, Rockford, IL) was dissolved in methylene chloride (1.0 mg/ml), and 1 ml was pipetted into a sterile, pyrogen-free 10 ml vial. The vial was rotated and dried under nitrogen to evaporate methylene chloride.

#### Anion Exchange Resin Filters

100- to 200-mesh AG1X8 anion exchange resin (Bio-Rad Labs, Richmond, CA) was washed using sterile, pyrogen free water. The anion exchange resin was aseptically loaded onto a 0.22 μm filter disc (1.1 to 1.5 gram wet resin/filter unit) (Millipore Corporation, Milford, MA). The resin was washed using the following solutions in a sequence: 10 ml sterile, pyrogen-free water; 10 ml sterile 0.1 N NaOH; 10 ml pyrogen free water; 10 ml 0.1 N sodium phosphate buffer (pH 7.4); and finally by 3.3 ml 0.1 N sodium phosphate buffer with 1% HSA.

#### Labeling process [65,66]

0.50 ml of HuCC49deltaC_H_2 antibody (1.5 mg/ml) was added to a 10 ml vial coated with 1 mg of iodogen. Then, 0.8 ml of phosphate buffered Na^124^I (150 MBq/ml) (IBA Molecular, Dulles, VA) was added to the vial. The reagents were allowed to react for 15 minutes. Free ^124^I was removed using an exchange resin filter disc. Then, 1 ml of 5% sucrose with 0.05% Tween 20 in saline was used to elute the labeled antibody. The purified ^124^I-HuCC49deltaC_H_2 was passed through a 0.22 mm Millipore filter (Millipore Corporation, Milford, MA) for *in vivo *applications. Radiolabeling efficiency was monitored using thin layer chromatography, which was performed on silica-gel-impregnated glass fiber sheets (Pall Corporation, East Hills, NY). 0.02 M citrate buffer (pH 5.0) was used as the mobile phase.

### Xenograft mouse model with human colon adenocarcinoma cells (LS174T)

The human colon adenocarcinoma cells, LS174T, were trypsinized for 2 minutes, collected, and washed with PBS under 1000 rpm × 2 minutes. The washed cells (5×10^6 ^cells) were resuspended in a mixture of 50 μl of PBS and 50 μl of matrigel medium (Invitrogen, Carlsbad, California) and then injected subcutaneously into the dorsal surface (back) of female athymic Nu/Nu nude mice (National Cancer Institute at Frederick, Frederick, MD) that were 4 to 6 weeks of age. The resultant LS174T tumor implants on the xenograft mice were allowed to grow for approximately two weeks, reaching a tumor implant volume of up to 300 mm^3^. The xenograft mice used in this preliminary study were not pretreated with an oral saturated solution of potassium iodide (SSKI).

### ^124^I-HuCC49deltaC_H_2 and ^18^F-FDG injections of the xenograft mice

Two xenograft mice were successfully injected intravenously (i.v.), by way of tail vein injection, with ^124^I -HuCC49deltaC_H_2, at a dose of 0.6 MBq and 0.75 MBq, respectively. Two additional xenograft mice were successfully injected intraperitoneally (i.p.) with ^124^I-HuCC49deltaC_H_2, at a dose of 1.4 MBq and 2.5 MBq, respectively. As a control, one xenograft mouse was successfully injected i.v., by way of tail vein injection, with 7.4 MBq of ^18^F-FDG. Pre-injection and post-injection blood glucose levels were not monitored in the xenograft mice.

*In vitro *binding studies with Cy7-labeled HuCC49deltaC_H_2 on LS174T cells, *in vivo *pharmacokinetics and biodistribution studies with Cy7-labeled HuCC49deltaC_H_2 in xenograft mice, and *ex-vivo *post-mortem biodistribution studies with Cy7-labelled HuCC49deltaC_H_2 on excised tumor implants and organs (i.e., spleen, kidney, lung, heart, liver, stomach, and intestine) from xenograft mice were previously performed and reported elsewhere [67]. These studies with Cy7-labeled HuCC49deltaC_H_2 were compared to results after nontreatment, Cy7 alone, Cy7-labeled nonspecific human IgG, Cy7-labeled murine CC49, and pretreatment with unlabeled murine CC49 prior to administration of Cy7-labeled HuCC49deltaC_H_2 [67].

### MicroPET tumor imaging of the xenograft mice

Selection of microPET imaging time points was based on historical data as well as the physical half-lives of ^18^F (110 minutes) and ^124^I (4.2 days). For ^18^F-FDG, the standard accepted injection to scan time for humans and small animals is approximately 60 ± 10 minutes [68-70]. For ^124^I-HuCC49deltaC_H_2 injected xenograft mice, an initial 1 hour time point for baseline microPET imaging was used, as well as a time range of delayed microPET imaging from 18 hours to 24 hours after administration of ^124^I-HuCC49deltaC_H_2 to allow for distribution, uptake, and clearance. At selected time points (ranging from approximately 1 hour to 24 hours after injection of ^124^I-HuCC49deltaC_H_2), the xenograft mice were anesthetized with i.p. Ketamine (100 mg/kg)/Xylazine (10 mg/kg) and then scanned on an Inveon microPET scanner (Siemens Medical Solutions, Knoxville, TN). Image acquisition and analysis were performed by using Inveon Acquisition Workplace (Siemens Medical Solutions, Knoxville, TN). Xenograft mice initially underwent a transmission scan with a cobalt-57 source for 402 seconds for attenuation correction and quantification. Xenograft mice then underwent a PET emission scan at approximately 1 hour, 18 hours, and 20 hours after injection of ^124^I-HuCC49deltaC_H_2 with an acquisition time of 400 seconds and again at approximately 23 hours or 24 hours after injection of ^124^I-HuCC49deltaC_H_2 with an acquisition time of 800 seconds. For the ^18^F-FDG injected xenograft mouse, a PET emission scan was obtained at approximately 50 minutes after injection of ^18^F-FDG with an acquisition time of 400 seconds. The energy window of all PET emission scans was set to 350 keV to 650 keV, with a time resolution of 3.4 ns. Each emission acquisition data set was attenuation corrected with the attenuation transmission scan taken of each individual mouse at each designated time point and arranged into sinograms. The resultant sinograms were iteratively reconstructed into three dimensional volumes using an ordered-subset expectation maximization (OSEM) reconstruction algorithm. The transmission acquisition yielded an approximation of body volume and anatomic localization, such that regions of interest could be created to represent portions of the mouse anatomy, specifically, whole body, the LS174T tumor implant, and a designated background area (i.e., left lower quadrant of the abdomen).

The region of interest (ROI), for determination of tumor implant volume, was drawn manually by qualitative assessment to cover the entire tumor implant volume by summation of voxels using the Inveon software (Siemens Medical Solutions, Knoxville, TN) in a manner similar to that previously published by Jensen *et al. *[70]. In the study by Jensen *et al*., they compared the accuracy of xenograft measurement by *in vivo *caliper measurement versus microCT-based and microPET-based measurement and found microCT to be the most accurate measurement method [70]. We used a similar method in conjunction with the transmission image to generate the tumor implant volume. PET activity within the volumetric ROI then yielded the resultant average intensity counts for the tumor implant and for the designated background area. Finally, to generate a quantification measurement value for the activity of ^124^I-HuCC49deltaC_H_2 and of ^18^F-FDG that was imaged on microPET within a given LS174T tumor implant, we utilized the unitless value of the relative ratio of the average intensity counts. This relative ratio of the average intensity counts was determined by dividing the average intensity counts from the tumor implant volume by the average intensity counts of the designated background area. We elected to generate this relative ratio of the average intensity counts as a quantification measurement value due to the fact that mouse body weights and tumor implant weights were not recorded and microCT was not obtained on all of the xenograft mice during the course of the current preliminary study.

### Statistical analysis

The software program IBM SPSS^® ^18 for Windows^® ^(SPSS, Inc., Chicago, Illinois) was used for the data analysis. One-way analysis of variance (ANOVA) was utilized for the comparison of the relative ratio of average intensity counts of the LS174T tumor implants.

## Results

After chromatographic purification, 98% of ^124^I was bound to the chelated HuCC49deltaC_H_2 antibody, as determined by thin layer chromatography. The radioactivity of ^124^I-HuCC49deltaC_H_2 obtained was 15 MBq/ml.

**Figure 1:**
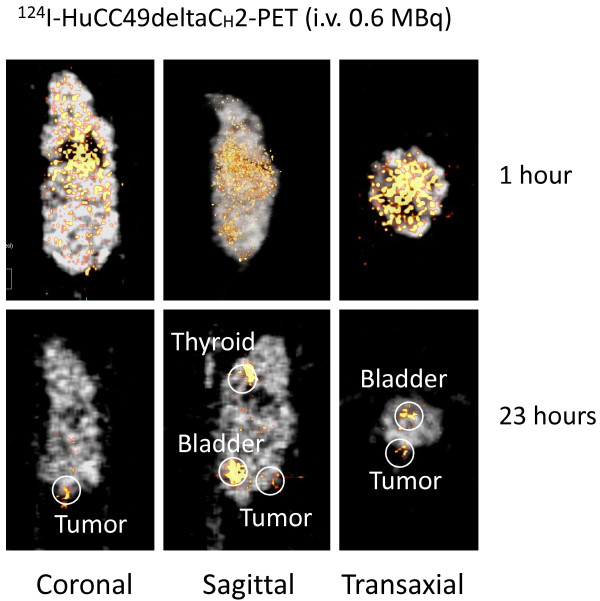
**Intravenous (i.v.) administration through the tail vein of 0.6 MBq of ^124^I-HuCC49deltaC_H_2 for microPET imaging of the LS174T xenograft mouse model**. MicoPET imaging is shown at approximately 1 hour and at 23 hours after injection in coronal, sagittal, and transaxial views. At 23 hours after i.v. injection, ^124^I-HuCC49deltaC_H_2 was found to have specifically accumulated within the LS174T tumor implant.

**Figure 2:**
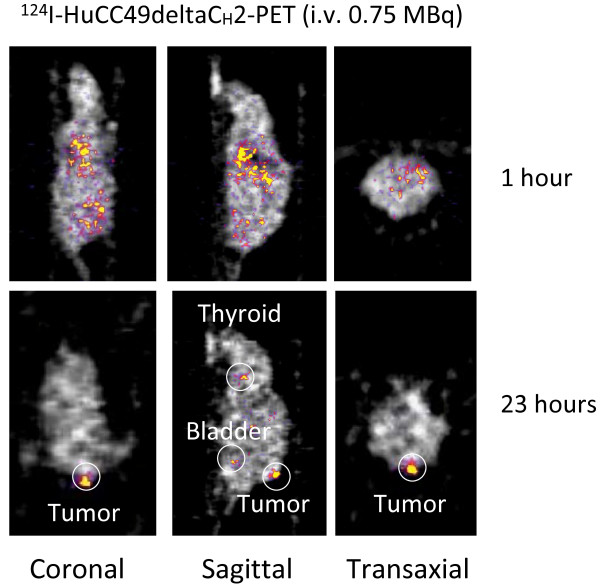
**Intravenous (i.v.) administration through the tail vein of 0.75 MBq of ^124^I-HuCC49deltaC_H_2 for microPET imaging of the LS174T xenograft mouse model**. MicoPET imaging is shown at approximately 1 hour and at 23 hours after injection in coronal, sagittal, and transaxial views. At 23 hours after i.v. injection, ^124^I-HuCC49deltaC_H_2 was found to have specifically accumulated within the LS174T tumor implant.

Figures 1 and 2 show the xenograft mice injected i.v. with ^124^I-HuCC49deltaC_H_2 at a dose of 0.6 MBq and 0.75 MBq, respectively. At approximately 1 hour after i.v. injection, ^124^I-HuCC49deltaC_H_2 was distributed within the systemic circulation, and demonstrated no significant localization within the LS174T tumor implants. At the time points of 18 hours and 23 hours after i.v. injection, ^124^I-HuCC49deltaC_H_2 was found to have specific localization within the LS174T tumor implants. The thyroid showed expected uptake of ^124^I, secondary to the lack of pre-treatment with SSKI. The bladder exhibited accumulation of ^124^I, indicating the degradation of ^124^I-HuCC49deltaC_H_2 and the excretion of free ^124^I into the urine.

**Figure 3:**
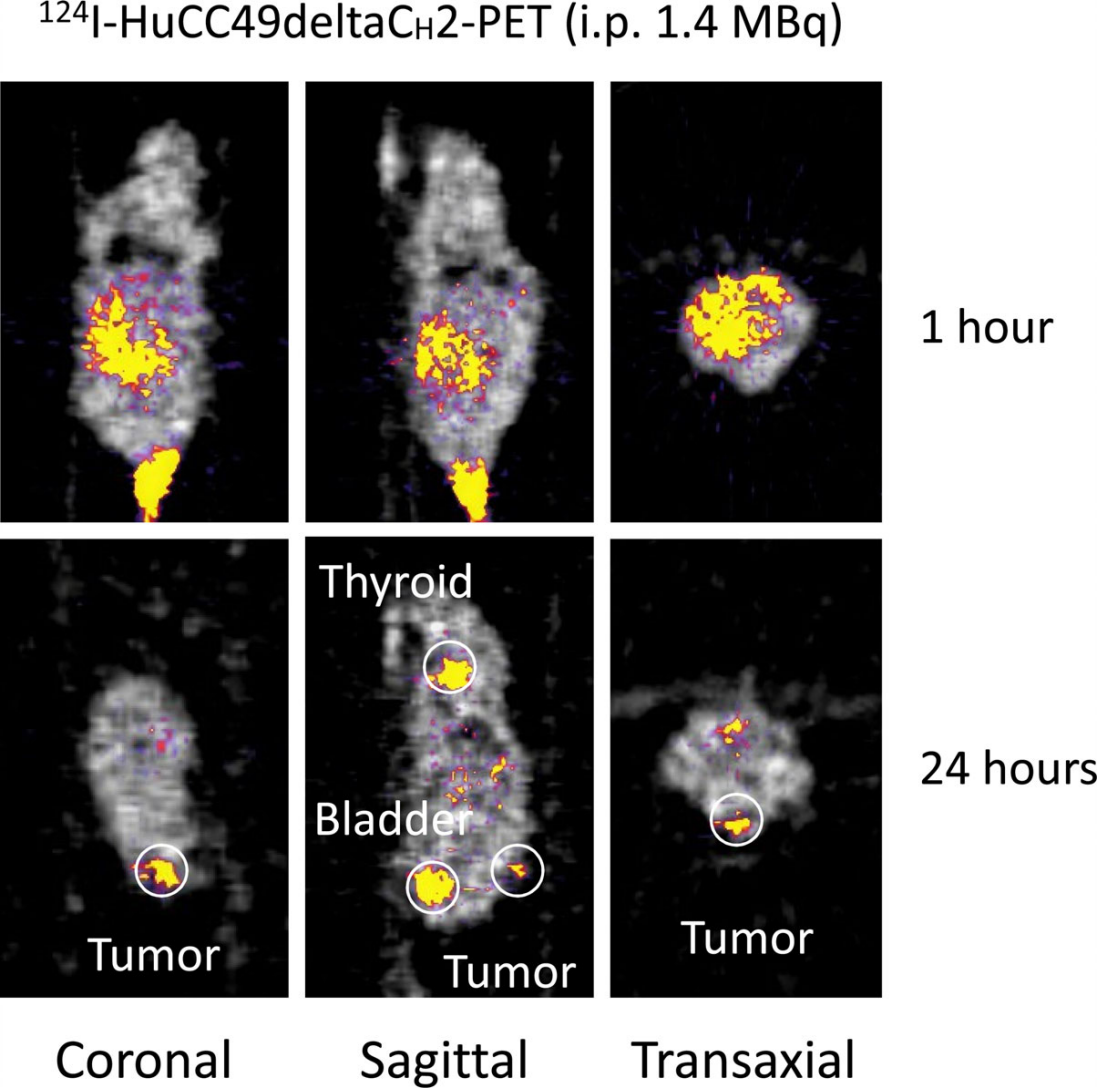
**Intraperitoneal (i.p.) administration of 1.4 MBq of ^124^I-HuCC49deltaC_H_2 for microPET imaging of the LS174T xenograft mouse model**. MicoPET imaging is shown at approximately 1 hour and at 24 hours after injection in coronal, sagittal, and transaxial views. At 24 hours after i.p. injection, ^124^I-HuCC49deltaC_H_2 was found to have specifically accumulated within the LS174T tumor implant. The area of increased activity (yellow) seen at the hind end of the mouse on the 1 hour coronal and sagittal images represents subcutaneous activity within the tail region due to previous failed tail vein injection. This subcutaneous activity within the tail region completely disappeared by the 24-hour image. Some nonspecific liver uptake is noted at 24 hours after i.p. administration of 1.4 MBq of ^124^I-HuCC49deltaC_H_2 secondary to use of the chelated form of the HuCC49deltaC_H_2 antibody.

**Figure 4:**
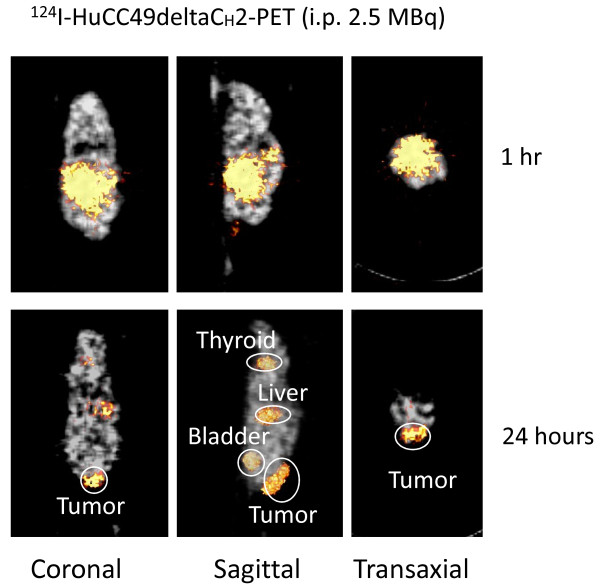
**Intraperitoneal (i.p.) administration of 2.5 MBq of ^124^I-HuCC49deltaC_H_2 for microPET imaging of the LS174T xenograft mouse model**. MicoPET imaging is shown at approximately 1 hour and at 24 hours after injection in coronal, sagittal, and transaxial views. At 24 hours after i.p. injection, ^124^I-HuCC49deltaC_H_2 was found to have specifically accumulated within the LS174T tumor implant. Significant nonspecific liver uptake was most pronounced at 24 hours after i.p. administration of 2.5 MBq of ^124^I-HuCC49deltaC_H_2 secondary to use of the chelated form of the HuCC49deltaC_H_2 antibody.

Figures 3 and 4 show the xenograft mice injected i.p. with ^124^I-HuCC49deltaC_H_2 at a dose of 1.4 MBq and 2.5 MBq, respectively. At approximately 1 hour after i.p. injection, ^124^I-HuCC49deltaC_H_2 was distributed only within the peritoneal cavity, and demonstrated no significant localization within the LS174T tumor implants. At the time points of 20 hours and 24 hours after i.p. injection, ^124^I-HuCC49deltaC_H_2 was found to have specific localization within the LS174T tumor implants. The thyroid showed expected uptake of ^124^I, secondary to the lack of pre-treatment with SSKI. The bladder exhibited accumulation of ^124^I, indicating the degradation of ^124^I-HuCC49deltaC_H_2 and the excretion of free ^124^I into the urine. ^124^I-HuCC49deltaC_H_2 was also observed to accumulate within the liver on the microPET images and was most pronounced at the time points of 20 hours and 24 hours after i.p. injection of ^124^I-HuCC49deltaC_H_2 at a dose of 2.5 MBq (Figure 4). This was presumed to be secondary to use of the chelated form of the HuCC49deltaC_H_2 antibody.

**Figure 5:**
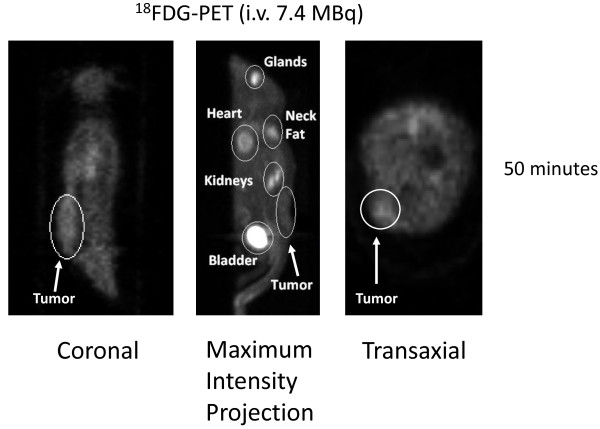
**Intravenous (i.v.) administration through the tail vein of 7.4 MBq of ^18^F-FDG for microPET imaging of LS174T xenograft mouse model**. MicroPET imaging is shown at approximately 50 minutes after injection in coronal, maximum intensity projection, and transaxial views. Only very weak ^18^F-FDG activity was noted within the LS174T tumor implant. In contrast, significant tumor-nonspecific ^18^F-FDG accumulation was noted in the heart, Harderian glands within the bony orbits of the eyes, brown fat of the posterior neck region, kidney, and bladder.

Figure 5 shows the xenograft mouse injected i.v. with 7.4 MBq of ^18^F-FDG and imaged by the microPET at approximately 50 minutes after injection. Multiple sites of tumor-nonspecific ^18^F-FDG accumulation were noted in the xenograft mouse. ^18^F-FDG was noted to avidly accumulate in the heart, the brown fat of the posterior neck, and the Harderian glands within the bony orbits of the eyes, all secondary to the high rate glucose metabolism within these tissues. ^18^F-FDG was noted to be rapidly eliminated from kidneys and bladder within 50 minutes after the i.v. injection. Only very minimal localization of ^18^F-FDG to the LS174T tumor implant was noted in the xenograft mouse model.

To generate a quantification measurement value for the activity of ^124^I-HuCC49deltaC_H_2 and of ^18^F-FDG that was imaged on microPET within a given LS174T tumor implant, we utilized the unitless value of the relative ratio of the average intensity counts, as determined by dividing the average intensity counts of the LS174T tumor implant by the average intensity counts of the designated background area. For comparing the localization of ^124^I-HuCC49deltaC_H_2 within the LS174T tumor implants at approximately 1 hour after i.v. and i.p. injection versus at 18 hours to 24 hours after i.v. and i.p. injection, the mean relative ratio of the average intensity counts was determined to be 0.34 (SD ± 0.29, range 0.06 to 0.64, n = 4) at approximately 1 hour after injection as compared to 2.58 (SD ± 0.99, range 1.57 to 4.57, n = 8) at 18, 20, 23, and 24 hours after injection (p = 0.001). This finding verifies a significantly increased level of specific localization of ^124^I-HuCC49deltaC_H_2 to LS174T tumor implants as compared to background tissues at 18 hours to 24 hours after injection. For comparing the localization of ^124^I-HuCC49deltaC_H_2 within the LS174T tumor implants for the i.v. injection route versus the i.p. injection route, the mean relative ratio of the average intensity counts was determined to be 2.31 (SD ± 0.71, range 1.83 to 3.356, n = 4) for the i.v. injection route at 18 and 23 hours after injection as compared to 2.85 (SD ± 1.26, range 1.57 to 4.57, n = 4) for the i.p. injection route at 20 hours and 24 hours after injection (p = 0.481), suggesting that i.v. and i.p. administration of ^124^I-HuCC49deltaC_H_2 achieved similar delivery efficiency. For comparing the localization of ^124^I-HuCC49deltaC_H_2 within the LS174T tumor implants at differing doses of ^124^I-HuCC49deltaC_H_2, the mean relative ratio of the average intensity counts was determined to be 2.03 (SD ± 0.14, range 1.93 to 2.13, n = 2) at the lowest dose administered (i.e., 0.6 MBq i.v.) as compared to 3.70 (SD ± 1.23, range 2.84 to 4.57, n = 2) at the highest dose administered (i.e., 2.5 MBq i.p.) (p = 0.195). Finally, for comparing the localization of ^124^I-HuCC49deltaC_H_2 versus ^18^F-FDG within the LS174T tumor implants, the mean relative ratio of the average intensity counts was 2.58 (SD ± 0.99, range 1.57 to 4.57, n = 8) for ^124^I-HuCC49deltaC_H_2 at 18, 20, 23, and 24 hours after i.v. and i.p. injection as compared to 1.05 (n = 1) for ^18^F-FDG at approximately 50 minutes after i.v. injection (p = 0.188). Although this demonstrates that there was 2.46 times greater localization of ^124^I-HuCC49deltaC_H_2 within the LS174T tumor implants as compared to ^18^F-FDG, this particular p-value did not reach statistical significance, and this is likely attributable to the statistic restraints of comparing only one time point for a single ^18^F-FDG injected xenograft mouse to that of 8 time points for 4 xenograft mice injected with ^124^I-HuCC49deltaC_H_2.

## Discussion

In the current preliminary report, ^124^I-HuCC49deltaC_H_2 demonstrated a significantly increased level of specific localization to LS174T tumor implants as compared to background tissues (p = 0.001) in the xenograft mouse model at 18 hours to 24 hours after injection as compared to at approximately 1 hour after injection. In contrast, in the same xenograft mouse model, ^18^F-FDG failed to demonstrate any increased level of specific localization to a LS174T tumor implant as compared to background tissues at approximately 50 minutes after injection. These findings, although based on a limited number of xenograft mice, re-enforce the recognized limitations of an ^18^F-FDG-based PET imaging strategy as compared to an antigen-directed and cancer-specific ^124^I-HuCC49deltaC_H_2-based PET imaging strategy.

In the current preliminary report, both i.v. and i.p. administration of ^124^I-HuCC49deltaC_H_2 resulted in specific localization on microPET imaging to the LS174T tumor implants in the xenograft mouse model at 18 and 23 hours and at 20 and 24 hours after injection, respectively, validating the use of both injection routes for use in preclinical animal studies evaluating ^124^I-HuCC49deltaC_H_2. Therefore, the end result of the transport of ^124^I-HuCC49deltaC_H_2 from the peritoneal cavity to the LS174T tumor implants after i.p. administration was similar to the transport of ^124^I-HuCC49deltaC_H_2 from the systemic circulation to LS174T tumor implants after i.v. administration. These results with ^124^I-HuCC49deltaC_H_2 are consistent with previous studies which have demonstrated the efficacy of i.p. administered anti-TAG-72 monoclonal antibodies in patients with colorectal cancer [71,72].

Overall, these preliminary results in the LS174T colon adenocarcinoma xenograft mouse model are very encouraging and lay the ground work for further investigations into the use of this antigen-directed and cancer-specific ^124^I-radiolabeled anti-TAG-72 monoclonal antibody conjugate in human clinical trials related to preoperative, intraoperative, and postoperative PET-based imaging strategies [73]. Such an approach that utilizes PET-based imaging in conjunction with ^124^I-HuCC49deltaC_H_2 is clinically feasible and could potentially have a significant impact upon the current management of colorectal cancer, as well as upon other TAG-72 antigen-expressing adenocarcinomas.

Despite the promising results of our current preliminary report that clearly show that the ^124^I-radiolabled anti-TAG-72 monoclonal antibody conjugate, ^124^I-HuCC49deltaC_H_2, shows high degree of specific localization to TAG-72 antigen expressing tumor implants in the xenograft mouse model, there are several shortcomings of our current experimental study design which led to non-optimization of our reported results and that will need to be further addressed in future experiments. These shortcomings are the small sample size, the lack of thyroid block by oral administration of SSKI, the use of the chelated form of the HuCC49deltaC_H_2 antibody, and the anesthetic and time constraints at the time of these preliminary experiments that did not allow for obtaining fused microPET/CT imaging of all the xenograft mice studied.

First, as is shown in Figures 1, 2, 3, and 4, significant thyroid uptake was seen on microPET imaging at the time points of 18 hours and 23 hours after i.v. injection and at the time points of 20 hours and 24 hours after i.p. injection of ^124^I-HuCC49deltaC_H_2. It has long been well-known in the nuclear medicine literature that if the thyroid is not blocked by the oral administration of SSKI, then resultant thyroid uptake of circulating radioactive iodine will freely occur [74-76]. This has been previously experimentally evaluated with radioiodine labeled anti-TAG-72 monoclonal antibodies [77]. As such, in the current animal experiments, the lack of thyroid blockade resulted in significant thyroid uptake of free ^124^I as the unbound ^124^I-HuCC49deltaC_H_2 was metabolized in the body and before the free circulating ^124^I was excreted into the urine. Therefore, pre-treatment of the xenograft mice with oral administration of SSKI to minimize thyroid uptake of free ^124^I would have resulted in more optimal microPET imaging, thus better illustrating our take-home message of specific localization of ^124^I-HuCC49deltaC_H_2 to LS174T tumor implants by minimizing the degree of thyroid localization of free ^124^I. This shortcoming was an oversight on our part and will be subsequently re-addressed in future xenograft mouse model experiments in which the xenograft mice are pretreated with oral SSKI.

Second, nonspecific liver uptake of ^124^I-HuCC49deltaC_H_2 was seen on microPET imaging. As best illustrated in Figure 4, significant nonspecific liver uptake was most pronounced at the time points of 20 hours and 24 hours after i.p. administration of the higher dose (2.5 MBq) of ^124^I-HuCC49deltaC_H_2. This nonspecific liver uptake was less intense on microPET imaging at the time points of 20 hours and 24 hours after i.p. administration of a lower dose (1.4 MBq) of ^124^I-HuCC49deltaC_H_2 (Figure 3) and was minimally present on microPET imaging at the time points of 18 hours and 23 hours after i.v. administration of either dose (0.6 MBq or 0.75 MBq) of ^124^I-HuCC49deltaC_H_2 (Figure 1 and Figure 2). A similar pattern of accumulation within the liver has been previously reported for various chelated radiolabeled CC49 monoclonal antibodies [78], as well as for a single-chain Fv version of the radiolabeled CC49 monoclonal antibody [79]. It has been suggested that the high accumulation of these radiolabeled monoclonal antibody in the liver is likely due to the metabolism of the chelated form of the antibody within the liver [78]. Clearance and metabolism of IgG antibodies occurs predominantly through the reticuloendothelial system (RES), primarily in the liver and spleen, which both contain Kupffer cells [78,79]. Furthermore, IgG antibodies are bound and internalized by asialoglycoprotein receptors in the liver cells, increasing the retention of IgG antibodies within the liver. Therefore, it is our contention that the nonspecific liver uptake of ^124^I-HuCC49deltaC_H_2 seen on microPET imaging is explainable by our use of chelated form of the HuCC49deltaC_H_2 antibody. It should be noted that our inadvertent use of the chelated form of the HuCC49deltaC_H_2 antibody was not recognized until after analysis of the microPET imaging, as is best exemplified at the time points of 20 hours and 24 hours after i.p. administration of 2.5 MBq of ^124^I-HuCC49deltaC_H_2. Therefore, use of the non-chelated form of the HuCC49deltaC_H_2 antibody would have potentially eliminated the nonspecific liver uptake of ^124^I-HuCC49deltaC_H_2, thus better illustrating our take-home message of specific localization of ^124^I-HuCC49deltaC_H_2 to LS174T tumor implants. This shortcoming was an oversight on our part and will be subsequently re-addressed in future xenograft mouse model experiments in which the non-chelated form of the HuCC49deltaC_H_2 antibody is utilized.

Third, at the time of this preliminary animal experiment, due to limitations in the type of anesthetic available (i.e., only i.p. Ketamine/Xylazine was available and inhalation isoflurane anesthesia was not available), due to the time constraints necessary for repetitive scanning in both a microPET and a microCT format, and due to the limited number of xenograft mice available, fused microPET/CT imaging was only obtained on one of the five xenograft mice. Therefore, while all five xenograft mice were imaged by the dedicated microPET scanner, only one xenograft mouse (i.v. injection of ^124^I-HuCC49deltaC_H_2 at a dose of 0.6 MBq) was also imaged with the microCT scanner at the time point of 24 hours after i.v. injection, thus allowing for reconstruction of fused microPET/CT images. In this particular case of fused microPET/CT imaging, the microCT images demonstrated relatively good correlation of anatomy with the transmission images and assisted in the accurate determination of tumor implant volume from the transmission scan. It is evident within the molecular imaging literature that fused-modality PET-based imaging is superior to PET alone-based imaging, both for the PET/CT platform and for the PET/MRI platform [73,80-83]. These fused imaging platforms can provide both molecular/functional information and structural information that can more accurately and more precisely localize various disease processes. It is our intention to subsequently re-address this shortcoming in future xenograft mouse model experiments by utilizing a fused microPET/CT imaging platform in all of the xenograft mice.

As a last notable point of discussion, some may contend that the lack of specific localization of ^18^F-FDG to the LS174T tumor implant as compared to the background tissues was the specific result of the type of anesthetic used for the Nu/Nu nude mice in the current preliminary study (i.e., i.p. Ketamine/Xylazine instead of inhalation isoflurane anesthesia). It has been previously reported that C57BL/6 mice injected with ^18^F-FDG and having received Ketamine/Xylazine anesthesia demonstrate increased blood glucose levels, as well as increased ^18^F-FDG activity within multiple normal tissues, such as in muscle, lung, liver, kidney, and blood, as compared to C57BL/6 mice injected with ^18^F-FDG that received no anesthesia [84,85]. It has been suggested by some authors that these metabolic effects are mediated through the inhibition of insulin release, and that such effects are most prominent in mice kept fasting for only 4 hours, but are substantially attenuated by 20 hours of fasting [84]. In our preliminary animal experiments, the xenograft mice were kept without food for approximately 14 hours prior to the injection of ^18^F-FDG and ^124^I-HuCC49deltaC_H_2. Therefore, the previously described metabolic effects resulting from only a short-duration fast should have been minimized. Furthermore, these same authors reported that Ketamine/Xylazine anesthesia did not significantly alter ^18^F-FDG activity within Lewis lung carcinoma (LLC) subcutaneous tumor implants on C57BL/6 mice as compared to the same scenerio with no anesthesia [84]. In contrast to Ketamine/Xylazine, low dose (0.5%) inhalation isoflurane anesthesia has been reported to resulted in no significant increase in ^18^F-FDG activity within normal tissues (i.e., muscle, lung, liver, and kidney) of C57BL/6 mice as compared to the same scenerio with no anesthesia [84,85]. These findings indirectly suggest that the use of low dose (0.5%) inhalation isoflurane anesthesia for the Nu/Nu nude mice in our current preliminary study could have potentially provided a means to eliminate any negative impact of the choice of anesthetic on the absolute level of ^18^F-FDG activity within the LS174T tumor implant and the various normal tissues. Based upon these findings, it is our plan to use inhalation isoflurane anesthesia in our future proposed animal studies in order to minimize the occurence of any such issues.

## Conclusions

On microPET imaging, ^124^I-HuCC49deltaC_H_2 demonstrates an increased level of specific localization to tumor implants of LS174T colon adenocarcinoma cells as compared to background tissues in the xenograft mouse model, while ^18^F-FDG failed to demonstrate this same finding. Clearly, a PET-based imaging approach that utilizes ^124^I-HuCC49deltaC_H_2 is feasible and could potentially have a significant impact upon the current management of colorectal cancer and other TAG-72 antigen-expressing adenocarcinomas. This antigen-directed and cancer-specific ^124^I-radiolabled anti-TAG-72 monoclonal antibody conjugate holds future potential for use in human clinical trials for preoperative, intraoperative, and postoperative PET-based imaging strategies, including fused-modality PET-based imaging platforms.

## Competing interests

SPP, NCH, GHH, RXX, CMM, and DS are all associated with Enlyton, Ltd. as non-salaried staff scientists and have equity in the company. EWM is associated with Enlyton, Ltd. as the non-salaried Chief Science Officer and has equity in the company. The authors declare that they have no other competing interests.

## Authors' contributions

PZ was involved in the study design, study execution, data collection and analyses, and writing and editing all aspects of this manuscript. SPP was involved in the study design, data collection and analyses, as well as the writing and editing of all aspects of this manuscript. NCH was involved in the study design, data analyses, as well as the writing and editing of all aspects of this manuscript. MMC performed all the PET imaging, data collection and analyses, and was involved in editing this manuscript. GHH was involved in the study design, performed the radiolabeling of the HuCC49deltaC_H_2 with ^124^I, and was involved in editing this manuscript. RXX was involved in the study design, study execution, and was involved in editing this manuscript. CMM and MAJ were involved in editing this manuscript. MVK and EWM were involved in the study design and in editing this manuscript. DS was involved in the study design, study execution, data collection and analyses, and writing and editing all aspects of this manuscript. All of the authors have read and approved the final version of this manuscript.
